# Infarct healing is a dynamic process following acute myocardial infarction

**DOI:** 10.1186/1532-429X-14-62

**Published:** 2012-09-02

**Authors:** Sean D Pokorney, José F Rodriguez, José T Ortiz, Daniel C Lee, Robert O Bonow, Edwin Wu

**Affiliations:** 1 Department of Medicine, Division of Cardiology, Bluhm Cardiovascular Institute, Northwestern University Feinberg School of Medicine, Northwestern Memorial Hospital, 676 Saint Clair St. Suite 600, Chicago, IL, 60611, USA; 2 Departments of Medicine and Radiology, Northwestern University Feinberg School of Medicine, 676 Saint Clair St. Suite 600, Chicago, IL, 60611, USA

**Keywords:** Acute coronary syndrome, Cardiovascular magnetic resonance, Infarct size, Late gadolinium enhancement, Remodeling

## Abstract

**Background:**

The role of infarct size on left ventricular (LV) remodeling in heart failure after an acute ST-segment elevation myocardial infarction (STEMI) is well recognized. Infarct size, as determined by cardiovascular magnetic resonance (CMR), decreases over time. The amount, rate, and duration of infarct healing are unknown.

**Methods:**

A total of 66 patients were prospectively enrolled after reperfusion for an acute STEMI. Patients underwent a CMR evaluation within 1 week, 4 months, and 14 months after STEMI.

**Results:**

Mean infarct sizes for the 66 patients at baseline (acute necrosis), early follow-up (early scar), and late follow-up (late scar) were 25 ± 17 g, 17 ± 12 g, and 15 ± 11 g, respectively. Patients were stratified in tertiles, based on infarct size, with the largest infarcts having the greatest absolute decrease in mass at early and late scar. The percent reduction of infarct mass was independent of initial infarct size. There was an 8 g or 32% decrease in infarct mass between acute necrosis and early scar (p < 0.01) with a 2 g or 12% additional decrease in infarct mass between early and late scar (p < 0.01).

**Conclusions:**

Infarct healing is a continuous process after reperfusion for STEMI, with greatest reduction in infarct size in the first few months. The dynamic nature of infarct healing through the first year after STEMI indicates that decisions based on infarct size, and interventions to reduce infarct size, must take into consideration the time frame of measurement.

## Background

The role of left ventricular (LV) remodeling in the clinical course of heart failure following an acute myocardial infarction (MI) is well recognized [1-4]. It is known that identification of previous MI, as determined by late gadolinium enhancement (LGE) cardiovascular magnetic resonance (CMR), has a significant impact on a patient’s mortality [5,6]. We have previously shown that initial infarct size by LGE on CMR, measured within a few days after reperfusion for ST-segment elevation MI (STEMI) is associated with cardiac event-free survival [7]. Other investigators have demonstrated that the area of LGE decreases within the first few months of the acute event [8-10]. This change was attributed to clearing of necrotic myocardium, inflammatory cells, residual edema, and hemorrhage and replacement by scar tissue.

It is unknown over what period of time infarct healing occurs from acute necrosis to chronic scar, as well as how the initial infarct size impacts the amount of healing and the degree of LV remodeling. We hypothesized that the majority of infarct healing occurs within 3–4 months of MI but continues at a slower rate for up to a year.

## Methods

We prospectively enrolled 66 patients admitted to the coronary care unit at Northwestern Memorial Hospital following an acute STEMI between March 1999 and July 2008. All patients underwent emergent percutaneous coronary intervention (PCI) and were willing to have CMR performed within 1 week and at approximately 4 months and 12 months after the event. Patients were included if they fulfilled the criteria of documented acute STEMI with [1] chest pain for more than 30 minutes, [2] at least 1.0 mm ST-segment elevation in two contiguous leads and [3] elevated creatinine phosphokinase MB (CPKMB) isoenzyme levels. We did not recruit patients with a known history of previous acute MI, PCI, or coronary artery bypass surgery. We also excluded patients with frequent and recurrent ventricular arrhythmias, unstable hemodynamics requiring intravenous inotropic agents, or contraindications to CMR such as pacemakers or defibrillators. Before the first CMR study, all participants gave written informed consent to the study protocol, which was approved by the Institutional Review Board at Northwestern University.

### Cardiac magnetic resonance

CMR was performed in a 1.5 T clinical scanner (Siemens, Erlangen, Germany). All images were acquired with a body phased-array receiver coil during repeat breath-holds and were electrocardiographically gated as previously described [11-14]. Functional assessment of the left ventricle was performed without contrast using a cine steady-state free precession (FISP) sequence. Sequential short axis images were acquired every 10 mm with a @6 mm slice thickness to cover the entire left ventricle. LGE images were acquired at identical slice positions as the cine images at least 10 minutes after the administration of gadopentetate dimeglumine (gadolinium-DTPA; Berlex, Montville, NJ, USA), 0.2 mmol/kg of body weight, using a T1-weighted, inversion-recovery, fast gradient-echo pulse sequence with inversion times set to null normal myocardium [13]. Matrix size was 256, resulting in a typical voxel size of 1.9 mm by 1.4 mm by 6.0 mm. All images were acquired using the same technique and pulse sequences to minimize variation in image quality that could impact infarct size quantification.

Quantitative analysis of LV mass, end-diastolic volume, end-systolic volume, and ejection fraction (EF) were performed by manually tracing the epicardial border (excluding epicardial fat) and endocardial borders (excluding papillary muscles) at end-diastole and end-systole for each short-axis slice. Three-dimensional volumes were calculated as the sum of [area * (slice thickness + distance between slices)] of all short axis slices. Volume indices were calculated by dividing the end-diastolic volume (EDVI) or end-systolic volume (ESVI) by the body surface area.

Acutely necrotic, early scar, and late scar tissue areas were determined by manually planimetering the regions of LGE (>2 standard deviations over remote signal intensity) on each of the contrast-enhanced CMR tomograms and summed, as previously described and performed by our group. [11-13]. Areas of microvascular obstruction (MVO) (dark areas of absent contrast surrounded by hyperenhanced infarct tissue on LGE images) were included in the total infarct area [5,15]. The summed area was multiplied by the specific gravity of myocardium to obtain the infarct mass. Infarct size was the percent of total LV mass that was infarct. In a subset of 56 studies including a mix of acute necrosis, early scar, and late scar studies, interobserver reproducibility was performed between two observers. The correlation coefficient for infarct size is 0.97 with a mean difference of 0.16% with a 95% confidence interval range from −3.6% to 3.9%.

### Statistical analysis

Results are expressed as mean ± standard deviation, unless otherwise noted. A paired t-test was used in the primary analysis to compare quantitative values between two time points (acute necrosis to early scar and early scar to late scar). Patients were further divided into tertiles by infarct size (small, medium, and large infarcts) for secondary analysis. Because of multiple comparisons, a Bonferroni correction was performed to avoid false positive findings. An alpha level of 0.05 was divided by 22 comparisons, and a two-tailed p-value of <0.002 was considered statistically significant. Additional repeated measure analysis of variance (ANOVA) analysis was performed to investigate differences in infarct mass and infarct size between tertile groups, followed by post-hoc testing with Bonferroni correction. Unpaired t-test and a Fisher’s exact test were used to compare differences between groups of continuous and categorical variables, respectively, to analyze infarct size and MVO on adverse LV remodeling.

## Results

### Baseline characteristics

The clinical and angiographic characteristics of the patients are shown in Table 1. The majority were men (91%) with MI involving the left anterior descending artery (56%). All subjects had successful reperfusion with PCI within 24 hours of presentation to the hospital. The patients were almost all discharged on appropriate medical therapy after PCI: anti-platelet agent, beta blocker, angiotensin converting enzyme inhibitor, and statin (see Table 1). The use of aldosterone antagonists was not collected.

**Table 1:** Baseline clinical characteristics for all subjects (n = 66)

**Risk factors**	
Age (years)	58±11
Male	60 (91%)
Diabetes mellitus	12 (18%)
Hypertension	28 (42%)
Hypercholesterolemia	36 (55%)
Family History	22 (33%)
Tobacco Use	32 (48%)
**Angiographic findings**	
Left anterior descending IRA	37 (56%)
Left circumflex IRA	7 (11%)
Right coronary IRA	22 (33%)
**Medication use**	
IIB-IIIA inhibitor	64 (97%)
Antiplatelet (aspirin and/or clopidogrel)	64 (97%)
ß-Blocker	62 (94%)
ACE inhibitor	57 (86%)
Statin	62 (94%)

Data presented as mean±SD or numbers (%) of subjects. ACE, angiotensin converting enzyme; CAD, coronary artery disease; IRA, infarct related artery; TIMI, thrombolysis in myocardial infarction.

### Infarct healing

**Figure 1:**
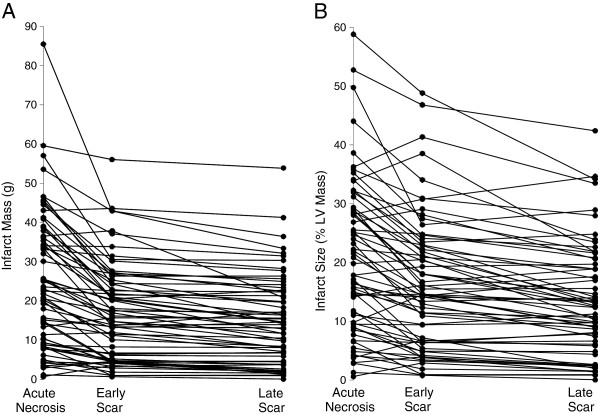
**Changes over time in infarct mass.** Scatter plot showing the changes in absolute infarct mass over time (**A**) and the changes in infarct size as a percent of left ventricular mass over time (**B**) for all patients.

Patients had CMR at a mean of 3 ± 1.7 days (acute necrosis), 4 ± 1.3 months (early scar), and 14 ± 3 months (late scar) (see Table 2). Infarct area, expressed both in infarct mass in absolute terms and as a percent of LV mass, decreased between the acute necrosis and early scar (25 ± 17 g vs 17 ± 12 g, p < 0.0001) and also between early and late scar (15 ± 11 g, p < 0.0001), with the greatest decrease in infarct size occurring between acute necrosis and early scar (Figure 1). There were 9 patients that had an increase in infarct size between early and late scar, meaning that 86% of patients had ongoing infarct healing beyond early scar. EDVI did not significantly change, but EF increased significantly between acute necrosis and early scar; increases in EF thereafter were not significant. LV mass decreased significantly between acute necrosis and early scar (from 115 ± 22 g to 103 ± 19 g, p < 0.0001). LV mass increased slightly, but not by a statistically significant amount, between early and late scar (from 103 ± 19 to 106 ± 22, p < 0.16). An increase in total LV mass could artificially decrease the percent of the LV with infarction without a true change in infarct size, but there was no statistically significant increase in total LV mass at either early or late scar. There is no linear relationship or correlation between changes in infarct mass and changes in LV mass.

**Table 2:** Cardiac Magnetic Resonance Results

	**Acute necrosis**	**Early scar**	**Late scar**	**p-value**	**p-value**
				**CMR 1-2**	**CMR 2-3**
Days after MI	3±1.7	125±40	436±93		
Infarct Mass (g)	25±17	17±12	15±11	< 0.0001	< 0.0001
Infarct Size (%)	21±13	16±11	13±9.5	< 0.0001	< 0.0001
EF (%)	42±10	46±10	46±11	< 0.0001	0.74
EDV Index (ml/m^2^)	79±17	82±18	82±20	0.04	0.78
LV Mass (g)	115±22	103±19	106±22	< 0.0001	0.16

Data presented as mean ± SD. EDV, end-diastolic volume; EF, ejection fraction; LV, left ventricular; MI, myocardial infarction. P-value < 0.002 statistically significant with Bonferroni correction (22 comparisons).

**Figure 2:**
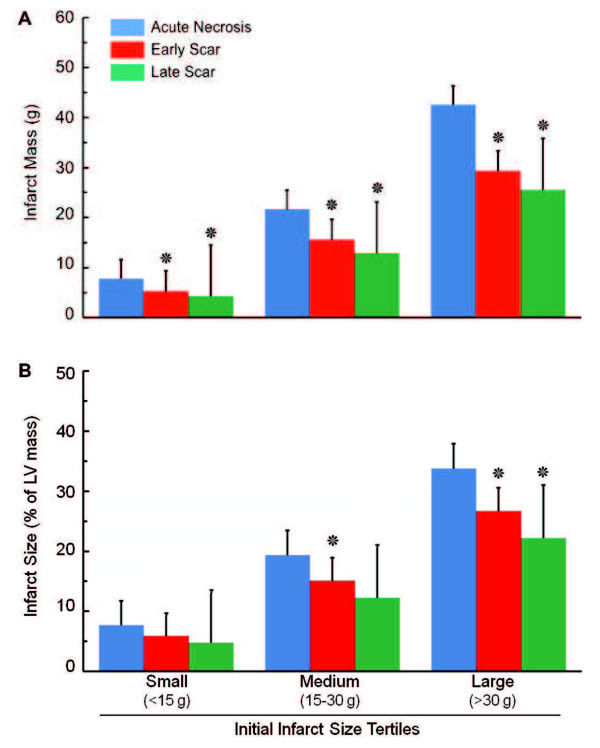
**Infarct healing over time.** Bar graph showing infarct mass (**A**) and infarct size (as percent of left ventricular mass, **B**) at acute necrosis, early scar, and late scar in patients subgrouped into tertiles of initial infarct size. Data are mean ± SD. Asterisks indicate p < 0.002 by paired t-test compared to previous time point, which is statistically significant after Bonferroni correction for 22 comparisons.

**Figure 3:**
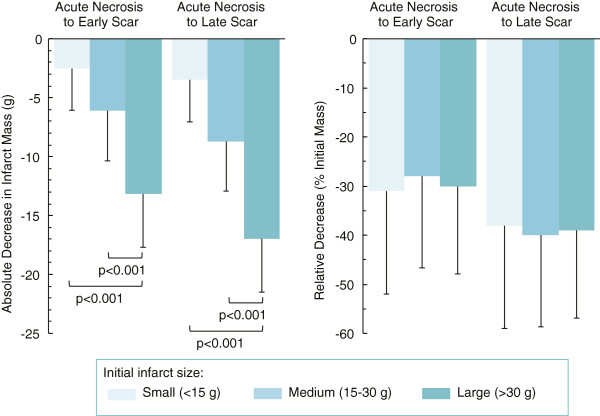
**Absolute versus relative changes in infarct size.** Bar graphs showing absolute decrease in infarct mass (left) and relative decrease as percent of the initial infarct mass (right) at the 3 time points among patients subgrouped into tertiles of initial infarct size. One-way ANOVA analysis with post hoc Bonferroni correction reveal that patients with the largest infarct size had the largest absolute decrease in infarct size when compared to the other two tertiles. However, the relative change in infarct size was not different between the infarct tertile groups.

When patients were subdivided into tertiles based on size of acute necrosis (Figure 2), the reduction in infarct size (both in terms of absolute mass and percent of LV mass) decreased significantly in patients with small (< 15 g), medium (between 15 g and 30 g), and large (> 30 g) infarcts at early scar and decreased further at late scar. The absolute reduction in infarct size was greatest in patients with the largest infarcts at baseline, with a reduction in infarct mass of 17 ± 10 g from acute necrosis to late scar, as compared to 3.5 ± 3.7 g in patients with the smallest infarcts (Figure 3). However, the relative reduction in infarct size over time (as a percent of the initial infarct size) was independent of initial infarct size and was the same across the 3 tertiles (Figure 3 right panel). The mean percent reduction in infarct mass among the tertiles ranged from 28% to 31% from acute necrosis to early scar and from 38% to 40% from acute necrosis to late scar.

MVO was identified in 44% of the cohort (29 of 66) in the setting of acute necrosis. Patients without MVO had an average infarct size of 16 ± 13 g, and patients with MVO had larger average infarct size of 35 ± 16 g (p < 0.0001). The MVO patients had a greater absolute reduction in infarct size of 9 ± 6 g over a year, but the relative decrease in size was not statistically different (p = 0.6) between patients with and without MVO.

### Ventricular remodeling

LV remodeling, defined as an increase in EDVI of greater than 20% following acute MI [16,17], was seen in 12 patients (18%) between acute necrosis and early scar and 14 patients (21%) between acute necrosis and late scar. For patients who developed adverse LV remodeling by 3 months, the mean infarct size decreased from 34 ± 24 g to 22 ± 15 g (p = 0.007), and for patients with remodeling at 14 months, the mean infarct size decreased from 30 ± 19 g to 19 ± 15 g (p = 0.002). Although patients with adverse remodeling had larger infarcts, there was no statistical difference in infarct sizes at any time point between patients with and without remodeling. Additionally, only 4 subjects with MVO had adverse LV remodeling and 10 subjects with MVO had no adverse LV remodeling (p = 0.2).

## Discussion

Infarct resorption occurs at a steady pace following the acute injury of an MI. In this study, we confirmed that 1) the percent reduction in infarct size is independent of the initial infarct size, and 2) infarct resorption occurs rapidly within the first few months following acute injury with more gradual reduction over the course of the next year.

Previous studies demonstrated that patients undergoing LV remodeling have a poor prognosis, and the likelihood of the heart undergoing adaptive adverse remodeling is dependent on the size of the acute MI [7,16,17]. Therefore, the goal of reperfusion therapies lies primarily in limiting the amount of acutely infarcted tissue. Little is known about the relation between the adaptive changes that the LV undergoes and infarct healing, as the acutely necrotic tissue is reabsorbed and replaced with a fibrotic collagenous scar. This process of remodeling is important clinically as more patients survive the acute MI, as many will later present with heart failure [18].

Reimer and Jennings alluded to the reduction of overall infarct size between acute and chronic studies in dogs. Earlier studies found that infarct size in dog models increased over the first 48 hours and then remained unchanged in size out to 10 days [19,20], but this data was published prior to wide use of inversion recovery technique for infarct analysis. Fieno and colleagues later described the natural history of healed reperfused and nonreperfused infarcts using more modern CMR techniques in dogs and found that infarct size decreased by 11 ± 8%, 33 ± 2%, and 46 ± 3% at 1 month, respectively, for arteries occluded for 45 minutes, for 90 minutes, and permanently. At 2 months, infarct size continued to decline by 10 ± 6%, 32 ± 2%, and 22 ± 3%, respectively, of the acute infarct size [10].

Studies in humans have reported similar findings. Ingkanisorn and colleagues demonstrated that the percent of infarcted myocardium decreased significantly in 20 patients, from 16 ± 12% to 11 ± 9% of the LV myocardium (reduction of 31%) over a median of 5 months after acute MI [9]. Lund and colleagues reported a decrease from 19 ± 10% to 14 ± 8% of LV myocardium (reduction of 26%) at 8 months post-MI [4]. In keeping with this prior experience, in the current study, we observed a reduction in infarct size from 21 ± 13% to 16 ± 11% of LV mass (reduction of 24%) over a mean 4-month time period.

With a greater number of patients, compared to the previous reports, and with a greater variability of initial infarct size, we demonstrated that the percent reduction of infarct size is independent of initial infarct size even though absolute reductions in infarct size are greater in patients with larger infarcts. Other investigators have noted that reductions in infarct size are largest in patients with CMR evidence for MVO [21]. However, we note that MVO tends to occur in subjects with larger infarct sizes.

The reports of changing infarct size over time has led some investigators to conclude that infarct size measured during the sub-acute phase overestimates the true infarct size [8,22]. Reimer and Jennings initially described a 24% “overestimation” of infarct size due to edema, but the edematous region was confined within the necrotic zone [8]. Aletras and colleagues [22] demonstrated differences in the extent of LGE between CMR contrast-enhanced T1-weighted and T2 weighted edema imaging. The T2-weighted edematous region matched the area at risk determined by microspheres, and infarct area was significantly smaller than the area at risk. However, if overestimation of initial infarct size due to edema or partial volume effects accounted for all of the apparent reductions in infarct size over time, reduction in the area LGE in the chronic phase should not occur. As demonstrated by Dall’Armellina, edema is unlikely to be present 6 months after AMI [23]. Our data indicate that incremental infarct healing occurs in the chronic phase, between 4 months and 14 months following acute MI.

The rapid reduction in infarct size over the first several months after MI has important implications regarding the clinical measurement of infarct size by CMR, as this is usually not performed immediately after MI (as in our research protocol). If infarct size measurement is performed within 3–4 months of an acute MI, the timing between the acute event and evaluation of scar is paramount. It is likely, although currently unproven, that infarct size decreases on a day-to-day or week-to-week basis, and more research is necessary to accurately characterize the minimum time frame during which CMR can measure decreases in the extent of LGE. Previous studies with myocardial perfusion imaging using single photon emission computed tomography (SPECT) reported differences in infarct size between 3 days and 1 week after MI [24]. It was hypothesized that the perfusion defects seen acutely were overestimations due to alterations in microvascular perfusion. SPECT imaging performed between 5 and 9 days after the acute event was thought to provide more accurate representation of the final infarct size [24]. Our data suggest that changes in perfusion sizes by SPECT imaging more likely represent a true reduction in infarct size.

Our data also indicate that infarct size continues to decrease slightly (3%) but significantly during the chronic phase between 4 and 14 months. Although the magnitude of this change may appear modest, emerging evidence suggests this finding may carry important prognostic and pathophysiologic implications. Kwong et al. assessed the prognostic value of infarct size by CMR in 195 patients with a clinical history of prior MI [25]. Although only 22% of patients had demonstrable scar by CMR, the presence and extent of LGE was significantly related to subsequent mortality. For every 1% increment in infarct size, there was an associated 10% increase in cardiac mortality (HR 1.10, [1.06 – 1.15], p < 0.0001). Roes et al. assessed the prognostic value of infarct size in 231 patients with evidence of LGE by CMR [26]. Infarct size was estimated using a semiquantitative scar score. In that study, a 1-point increment in scar score (equivalent to 1.5% of the left ventricle) was associated with a greater than 6-fold increase in all-cause mortality (HR 6.2 [1.7-23], p < 0.006), even after adjusting for 8 other clinical predictors including age, LVEF, and LV volumes.

## Conclusions

Reduction in infarct size is a dynamic and continuous process that extends well beyond the initial phase of infarct healing. Absolute changes in infarct size and mass beyond 4 months are small but statistically significant and may be clinically important. As more investigators use CMR imaging to monitor infarct size, a greater understanding of infarct healing will be achieved. Our data indicate that management decisions based on infarct size and interventions to reduce infarct size must take into consideration the time frame of measurement.

## Abbreviations

LV, Left ventricular; MI, Myocardial infarction; CMR, Cardiovascular magnetic resonance; STEMI, ST-segment elevation myocardial infarction; LGE, Late gadolinium enhancement; PCI, Percutaneous coronary intervention; EDVI, End diastolic volume index; ESVI, End systolic volume index; MVO, Microvascular obstruction; SPECT, Single photon emission computed tomography; ICD, Implantable cardioverter-defibrillator.

## Competing interests

There are no competing interests to disclose among the authors

## Authors’ contributions

SP: Analysis and interpretation of data, drafting of manuscript, critical revision and final approval of the manuscript. JR: Analysis and interpretation of data, critical revision and final approval of the manuscript. JO: Analysis and interpretation of data, critical revision and final approval of the manuscript. DL: Analysis and interpretation of data, critical revision and final approval of the manuscript. RB: Conception and design, analysis and interpretation of data, critical revision and final approval of the manuscript. EW: Conception and design, analysis and interpretation of data, critical revision and final approval of the manuscript. All authors read and approved the final manuscript.
